# Novel Cobalt Dichloride Complexes with Hindered Diphenylphosphine Ligands: Synthesis, Characterization, and Behavior in the Polymerization of Butadiene

**DOI:** 10.3390/molecules24122308

**Published:** 2019-06-21

**Authors:** Giovanni Ricci, Giuseppe Leone, Ivana Pierro, Giorgia Zanchin, Alessandra Forni

**Affiliations:** 1 CNR-Istituto per lo Studio delle Macromolecole (ISMAC), via A. Corti 12, 20133 Milano, Italy; giuseppe.leone@ismac.cnr.it (G.L.); ivana.pierro@ismac.cnr.it (I.P.); giorgia.zanchin@ismac.cnr.it (G.Z.); 2 CNR-Istituto di Scienze e Tecnologie Molecolari (ISTM), via C. Golgi 19, 20133 Milano, Italy; alessandra.forni@istm.cnr.it

**Keywords:** polymerization, cobalt complexes, catalyst, polybutadiene, X-ray structure

## Abstract

Two novel cobalt diphenylphosphine complexes were synthesized by reacting cobalt(II) chloride with *tert*-butyl(diphenyl)phosphine (P*^t^*BuPh_2_) and (*S*)-(+)neomenthyldiphenylphosphine [(*S*)-NMDPP]. The crystal structure of the former was determined by single-crystal X-ray diffraction studies. The two complexes were then used in combination with methylaluminoxane (MAO) for the polymerization of 1,3-butadiene: crystalline highly syndiotactic 1,2 poly(1,3-butadiene)s were obtained, with a 1,2 content and a syndiotactic index (percentage of syndiotactic triads [*rr*]) up to 95% and 85%, respectively. The results obtained further support and confirm what was already observed in the polymerization of 1,3-butadiene with CoCl_2_(PRPh_2_)_2_−MAO (R = methyl, ethyl, *normal*-propyl, *iso*-propyl, and cyclohexyl): the nature of the phosphine ligand strongly affects the polymerization stereoselectivity, the polymer syndiotacticity increasing with increasing phosphine ligand steric hindrance.

## 1. Introduction

Cobalt catalysts are well-known in the field of the stereospecific polymerization of 1,3-dienes, and they can perhaps be considered the most versatile catalytic systems among the various transition metal and lanthanide catalysts examined, since, depending on the catalytic formulation, they are able to provide all the possible polybutadiene stereoisomers: *cis*-1,4 polybutadiene, 1,2 polybutadiene, polybutadiene with a mixed *cis*-1,4/1,2 structure, and *trans*-1,4 polybutadiene [1,2,3,4,5,6].

Specifically, the catalytic systems obtained by combining CoCl_2_(PRPh_2_)_2_ (R = methyl, ethyl, *normal*-propyl, *iso*-propyl, and cyclohexyl) with methylaluminoxane (MAO) gave 1,2 polymers from butadiene and other terminally substituted 1,3-butadienes such as 1,3-pentadiene, 3-methyl-1,3-pentadiene, 1,3-hexadiene, 5-methyl-1,3-hexadiene, 1,3-heptadiene and 1,3-octadiene, simultaneously exhibiting a very high catalytic activity [7,8,9,10,11]. The tacticity of the 1,2 polymers obtained (i.e., the polymerization stereoselectivity) was found to depend on the type of phosphine ligand coordinated to the cobalt atom and on the monomer structure [12,13,14,15,16]: specifically, the syndiotacticity (i.e., the percentage of syndiotactic triads [*rr*%]) increased with increasing steric hindrance of the phosphine ligand, and the same obviously applies to the melting point of the polymers obtained.

**Figure 1:**
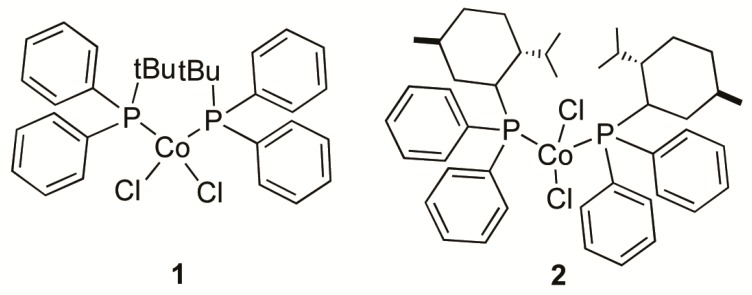
Bis-diphenylphosphine cobalt complexes used in this work.

In order to verify the above-described behavior (i.e., the increasing of syndiotacticity and melting point with increasing the ligand hindrance), and to obtain a syndiotactic 1,2polybutadiene having a melting point even closer to that of the polymer currently industrially produced by means of the Co(acac)_3_/AlEt_3_/H_2_O/CS_2_ system [17,18,19,20,21,22], we synthesized two novel cobalt complexes (**1** and **2** in Figure 1) by reacting cobalt dichloride with the following highly hindered phosphine ligands: *tert*-butyl(diphenyl)phosphine (P*^t^*BuPh_2_) and (*S*)-(+)neomenthyldiphenylphosphine [(*S*)-NMDPP]. The molecular structure of complex **1** was determined by X-ray diffraction, and then both complexes **1** and **2** were used in association with MAO for the polymerization of 1,3-butadiene. The results obtained clearly confirmed and supported the trend observed in the polymerization of 1,3-butadiene with the CoCl_2_(PRPh_2_)_2_−MAO catalyst systems (R = methyl, ethyl, *normal*-propyl, *iso*-propyl, and cyclohexyl) [8,9], and highly syndiotactic 1,2 polybutadienes were obtained, with a 1,2 content and a syndiotactic index (percentage of syndiotactic triads [*rr*]) up to 95% and 85%, respectively.

## 2. Results and Discussion

### 2.1. Synthesis and Characterization of Cobalt Complexes

**Figure 2:**
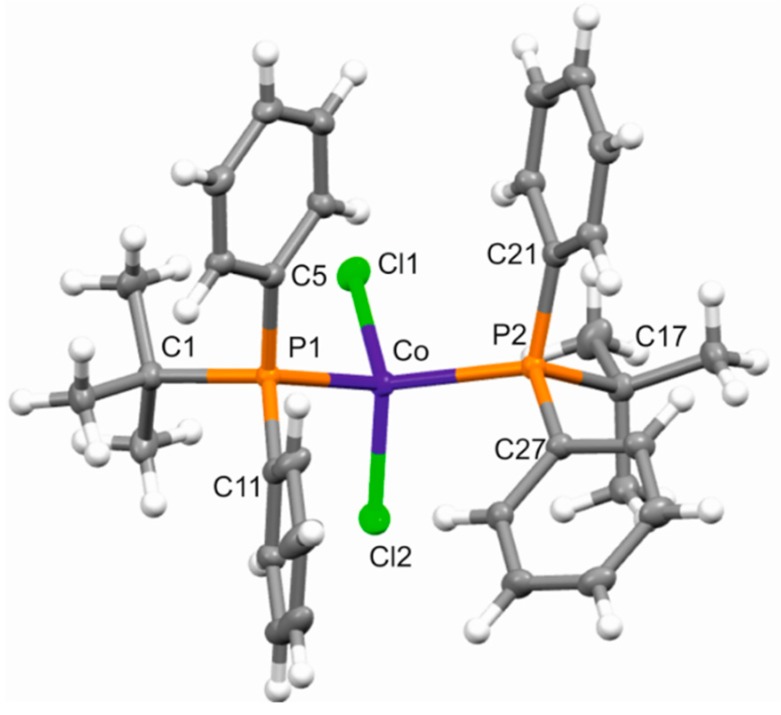
Molecular structure of **1** with thermal ellipsoids drawn at 50% probability level.

The two novel cobalt complexes were prepared according to a general experimental procedure already reported in the literature [23]. CoCl_2_ was dissolved in ethanol and reacted with an excess of phosphine. The solutions so obtained were kept under stirring at room temperature for one day, then the solvent was removed under vacuum. The residues were washed with cold pentane, then dried again under vacuum. Crystalline products were obtained by continuous extraction of the residues with boiling pentane. Single crystals suitable for X-ray diffraction studies were obtained for complex **1**, allowing its molecular structure to be determined (Figure 2). Selected bond lengths and angles are reported in Table 1. 

The molecular structure of **1** can be conveniently discussed together with those of the previously reported related CoCl_2_(PRPh_2_)_2_ complexes with R = ethyl (Et), *normal*-propyl (*^n^*Pr), and *iso*-propyl (*^i^*Pr) [8,9]. In fact, in these compounds, the phosphorus atoms bear the same aromatic groups (two phenyl rings) but different aliphatic substituents, going from small (Et, *^n^*Pr) to larger (*^i^*Pr, *^t^*Bu) groups. As expected, a clear differentiation between bonds connecting phosphorus and aliphatic (P−C_aliph_) or aromatic carbons (P−C_ar_) was found, the former being on average longer than the latter. Moreover, they both depended on the bulkiness of the aliphatic substituents, increasing from the complexes with *R* = Et and *^n^*Pr to **1**. Greater variation was clearly observed for the P−C_aliph_ distances, which measured on average 1.829(2), 1.827(2), 1.850(2), and 1.888(1) Å for *R* = Et, *^n^*Pr, *^i^*Pr, and *^t^*Bu, respectively. On the other hand, the average P–C_ar_ bond lengths showed a modest increase from 1.814(3) (both Et and *^n^*Pr) and 1.816(2) (*^i^*Pr) to 1.826(1) Å for ^t^Bu. The greater steric hindrance of the *^t^*Bu group also implies larger Co−P and Co−Cl distances in **1** than those observed in the other three complexes. In particular, the average Co–P bond lengths, measuring 2.370(1), 2.380(1), and 2.363(1) Å in the complexes with *R* = Et, *^n^*Pr, and *^i^*Pr, respectively, became as large as 2.433(1) Å in **1.**

Note that the different dimensions of the aliphatic substituents could explain the different conformations observed in complexes with *R* = *^t^*Bu and *^i^*Pr with respect to those of *R* = *^n^*Pr and Et derivatives. While in the former the aliphatic groups were in *trans* along the P−Co−P−*R* sequence, in the latter they were in *gauche* along the same sequence. This involves a different arrangement of the phenyl rings, which were both *gauche* in the complexes with *R* = *^t^*Bu and *^i^*Pr, granting a more efficient accommodation of the entire phosphinic group.

The observed difference in the conformations of CoCl_2_(P*R*Ph_2_)_2_ complexes may in turn account for the significantly larger P–Co–P angle for the derivatives with bulkier aliphatic groups. This angle in fact decreased from 113.69(1)° (*R* = *^t^*Bu) to 110.64(3)° (*^i^*Pr), 104.85(4)° (*^n^*Pr), and 102.89(3)° (Et). Widening the P−Co−P angle allows for a partial reciprocal distancing of the phenyl rings of different phosphines, which in *R* = *^t^*Bu and *^i^*Pr derivatives happened to be in pairs in a face-to-face repulsive arrangement, only partially relieved by a partial rotation around the P−C_ar_ bonds. The other bond angles were instead almost unchanged in the series of examined structures.

### 2.2. Polymerization of 1,3-Butadiene

The results obtained in the polymerization of 1,3-butadiene with the two new cobalt complexes based catalysts are shown in Table 2; the results obtained with CoCl_2_(PRPh_2_)_2_−MAO (R = *iso*-propyl, cyclohexyl) [8,9], which were found to be the systems providing 1,2 poly(1,3-butadiene) with the highest syndiotactic content, are added for comparison.

**Figure 3:**
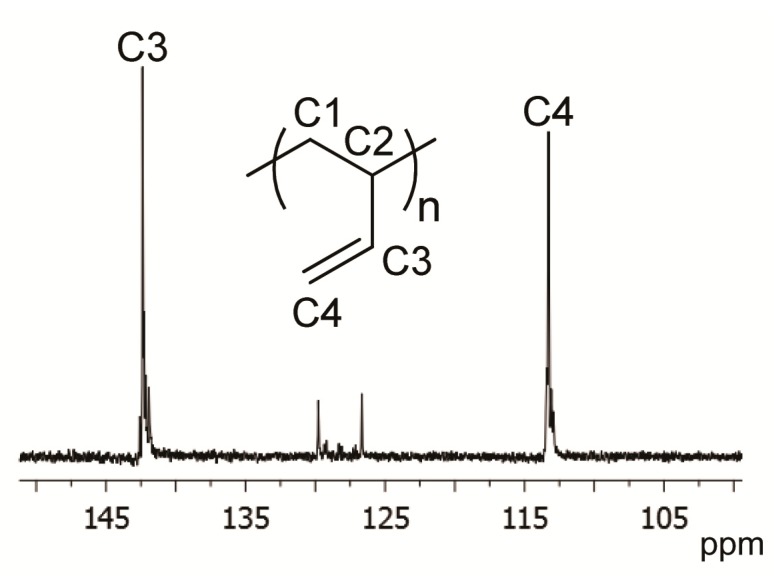
^13^C-NMR spectrum (olefinic region) of polybutadiene of Table 2, run 2.

With a MAO/Co molar ratio in the range 10−100, the CoCl_2_(P*^t^*BuPh_2_)_2_−MAO and CoCl_2_[PPh_2_(NMDPP)]_2_−MAO systems both gave polybutadienes with a predominantly 1,2 structure (up to 94.5%) and a syndiotactic content up to 85.5%. The ^13^C-NMR spectrum of the polybutadiene obtained with the system CoCl_2_(P*^t^*BuPh_2_)_2_−MAO at MAO/Co = 25, 0 °C, and in heptane as solvent (Table 2, run 4) is shown in Figure 3; the ^13^C NMR spectra of the other polybutadienes of Table 2 (Figures S1–S6), together with the FTIR spectra of the same polymers (Figures S7–S13) are reported in the Supplementary Materials. The 1,2 content and the syndiotacticity degree seemed to be only slightly affected by the MAO/Co molar ratio (in the range examined) (*cfr*. runs 1−2), whereas they seemed to be more influenced by the nature of the solvent (*cfr*. runs 2−3 and 5−6) and the polymerization temperature in particular (*cfr*. runs 3–4 and 6–7). This behavior was already observed for the analogous systems CoCl_2_(PRPh_2_)_2_−MAO (R = methyl, ethyl, *normal*-propyl, *iso*-propyl, and cyclohexyl), and a plausible interpretation for this behavior has already been given [8,9].

The syndiotactic degrees of the polybutadienes obtained with the two systems CoCl_2_(P*^t^*BuPh_2_)_2_−MAO and CoCl_2_[PPh_2_(NMDPP)]_2_−MAO were higher than those exhibited by CoCl_2_(P*^i^*PrPh_2_)_2_−MAO and CoCl_2_(PCyPh_2_)_2_−MAO (*cfr*. runs 1,8 and 5,9), which, as mentioned above, in the series CoCl_2_(PRPh_2_)_2_−MAO (R = methyl, ethyl, *normal*-propyl, *iso*-propyl, and cyclohexyl) were those giving the highest syndiotactic content. Therefore, the results obtained with the two novel systems described in the present paper confirm once more that the steric hindrance of the ligand exerts a strong influence on the mutual orientation of the allylic unit of the growing chain and of the new incoming monomer that is responsible for the polymerization stereoselectivity.

All the obtained polybutadienes were semicrystalline (the X-ray powder spectra are reported in the Supplementary Materials, Figures S14−S18), with a melting point in the range from 141 to 161 °C, and the crystallinity degree essentially depending on the polymer syndiotacticity.

## 3. Materials and Methods

### 3.1. General Procedure and Materials

*Tert*-butyl(diphenyl)phosphine (P*^t^*BuPh_2_) (Aldrich, St. Louis, MO, USA, 97%), (*S*)-(+)neomenthyldiphenylphosphine [(*S*)-NMDPP] (Strem Chemicals, Newburyport, MA, USA, 98%), anhydrous cobalt dichloride (Aldrich, 99.9%), and MAO (Aldrich, 10 wt.% solution in toluene) were used as received. Ethyl alcohol (Aldrich, ≥99.8%) was degassed under vacuum, then by bubbling dry dinitrogen and kept over molecular sieves; pentane (Aldrich, ≥99.5%) was refluxed over Na/K alloy for ca. 8 h, then distilled and stored over molecular sieves under dry dinitrogen; toluene (Aldrich, ≥99.5% pure) was refluxed over Na for ca. 8h, then distilled and stored over molecular sieves under dry dinitrogen. 1,3-Butadiene (Aldrich, ≥99.5%) was evaporated from the container prior to each run, dried by passing through a column packed with molecular sieves and condensed into the reactor which had been precooled to −20 °C. All the phosphine cobalt complexes were synthesized as indicated below, following a general procedure already reported in the literature [23].

### 3.2. Synthesis of Cobalt Phosphine Complexes

#### 3.2.1. CoCl_2_(P*^t^*BuPh_2_) (**1**)

Cobalt dichloride (CoCl_2_) anhydrous (1.24 g, 9.6 mmol) and 40 mL of ethanol were placed in a 250 mL flask: the blue solution obtained was kept under stirring, at 22 °C, for about 2 hours. Subsequently, *tert*-butyldiphenylphosphine (P*^t^*BuPh_2_) (5.1 g, 21 mmol) dissolved in ethanol (30 mL) was added: everything was kept under stirring, at room temperature, for 24 hours and subsequently the solvent was removed almost completely under vacuum. Then, pentane (40 mL) was added and the obtained suspension was kept under stirring at room temperature for 2 hours: at the end, the light blue/blue suspension obtained was filtered, the residue obtained was washed further with cold pentane (2 × 10 mL) and then dried under vacuum at room temperature. The blue solid obtained was then continuously extracted with boiling pentane; crystals of CoCl_2_(P*^t^*BuPh_2_)_2_ were formed directly on the bottom of the Schlenk tube during the extraction and further crops of crystals were obtained by cooling the supernatant pentane solution at −30 °C.

Yield, 4.71 g, 80% conversion based on the loaded cobalt dichloride. Elementary analysis [found (calculated) for C_32_H_38_Cl_2_CoP_2_, m.w. 614,43]: C: 62.50% (62.55%); H: 6.20% (6.23%); Cl: 11.50% (11.54%); P: 10.00% (10.08%); Co: 9.50% (9.59%).

The crystallographic data obtained are shown in Table 3. The FTIR spectrum of **1** is shown in Figure S19 of the Supplementary Materials.

#### 3.2.2. CoCl_2_[PPh_2_(NMDPP)]_2_ (**2**)

Cobalt dichloride (CoCl_2_) anhydrous (0.182 g, 1.4 mmol) and 20 mL of ethanol were placed in a 100 mL flask: the blue solution obtained was kept under stirring at room temperature for 1 hour. Subsequently, (*S*)-(+)neomenthyl-diphenylphosphine [(*S*)-NMDPP] (1.0 g, 3.08 mmol) dissolved in ethanol (30 mL) was added: the solution obtained was brought to 60 °C and kept under stirring at this temperature for 24 hours. The solvent was removed under vacuum, then pentane (30 mL) was added and the suspension was kept under stirring at room temperature for 2 hours. At the end, the light blue/blue suspension obtained was filtered and the residue obtained was further washed with pentane (2 × 10 mL) and then dried under vacuum at room temperature. Yield, 0.950 g of a light blue powder, 87% conversion with respect to the loaded cobalt dichloride.

Elementary analysis [found (calculated) for C_44_H_58_Cl_2_CoP_2_, m.w. 778,72]: C: 67.60% (67.86%); H: 7.40% (7.51%); Cl: 8.90% (9.11%); P: 7.90% (7.96%); Co: 7.50% (7.57%).

The FTIR spectrum of **2** is shown in Figure S20 of the Supplementary Materials.

### 3.3. X-ray Crystallographic Studies

A summary of the experimental details concerning the X-ray diffraction study of **1** is reported in Table 3. The crystals used for data collection were entirely covered with perfluorinated oil to reduce crystal decay. X-ray data were collected on a Bruker Smart Apex CCD area detector (Bruker AXS Inc., Madison, WI, USA) equipped with fine-focus sealed tube operating at 50 kV and 30 mA, using graphite-monochromated Mo Kα radiation (λ = 0.71073 Å). Data reduction was made using SAINT programs [24]; absorption corrections based on multiscan were obtained by SADABS [24]. The structures were solved by SHELXS-97 [25] and refined on F2 by full-matrix least-squares using SHELXL-14 [26]. The program ORTEP-III [27] was used for molecular graphics.

### 3.4. Polymerization

All operations were carried out under an atmosphere of dry dinitrogen. A standard procedure is reported. 1,3-Butadiene was condensed into a 25-mL dried glass reactor kept at −20 °C, then solvent was added and the obtained solution was brought to the desired polymerization temperature. MAO and the cobalt compound were then added as toluene solutions in the order given. The polymerization was terminated with methanol containing a small amount of hydrochloric acid, the polymer was coagulated and repeatedly washed with methanol, and then dried in vacuum at room temperature.

### 3.5. Polymer Characterization

^13^C-NMR and ^1^H-NMR measurements were performed with a Bruker AM 400 instrument (Bruker Italia Srl, Milano, Italy). The spectra were obtained in C_2_D_2_Cl_4_ at 103 °C (hexamethyldisiloxane, HMDS, as internal standard). The concentration of polymer solutions was about 10 wt.%. The polymer microstructure was determined as reported in the literature [28,29]. Differential scanning calorimetry (DSC) scans were carried out on a Perkin Elmer Pyris 8000 (Waltham, MA, USA). Typically, ca. 10 mg of polymer was analyzed in each run, while the scan speed was 20 °C/min under a dinitrogen atmosphere. Wide-angle X-ray diffraction (XRD) experiments were performed at 25 °C under nitrogen flux, using a Siemens D-500 diffractometer equipped with Soller slits (2°) placed before samples, 0.3° aperture and divergence windows, and a VORTEX detector with extreme energy resolution specific for thinner films. Cu Kα radiation with 40 kV × 40 mA power used was adopted, and each spectrum was carried out with steps of 0.05° 2θ, and 6 s measure time. FTIR spectra were acquired using a Perkin-Elmer (Waltham, MA, USA) Spectrum Two in attenuated total reflectance (ATR) mode in the spectral range of 4000–500 cm^−1^. The average molecular weight (*M*_w_) and the molecular weight distribution (*M*_w_/*M*_n_) were obtained by a high-temperature Waters GPCV2000 (Milford, MA, USA) size exclusion chromatography (SEC) system equipped with a refractometer detector. The experimental conditions consisted of three PL Gel Olexis columns, *ortho*-dichlorobenzene (*o*-DCB) as the mobile phase, 0.8 mL/min flow rate, and 145 °C temperature. The calibration of the SEC system was constructed using eighteen narrow *M*_w_/*M*_n_ poly(styrene) standards with *M*_w_s ranging from 162 to 5.6 × 10^6^ g mol^−1^. For SEC analysis, about 12 mg of polymer was dissolved in 5 mL of *o*-DCB with 0.05% of BHT as antioxidant.

## 4. Conclusions

We synthesized and characterized two novel cobalt diphenylphosphine complexes which, in combination with MAO, gave from 1,3-butadiene highly syndiotactic 1,2 polymers. The results obtained were as expected on the basis of the mechanistic hypotheses previously formulated [12,13], confirming their validity.

## Figures and Tables

**Table 1:** Selected bond lengths (Å) and angles (°) for CoCl_2_(P*^t^*BuPh_2_)_2_ (**1**).

Bond Lengths		Bond Angles	
Co–Cl1	2.2415(4)	Cl1–Co–Cl2	112.41(2)
Co–Cl2	2.2298(4)	P1–Co–P2	113.69(1)
Co–P1	2.4368(4)	C1–P1–C5	104.17(6)
Co–P2	2.4300(4)	C1–P1–C11	105.68(6)
P1–C1	1.8870(13)	C17–P2–C2	106.34(6)
P2–C17	1.8886(14)	C17–P2–C27	103.12(6)
P1–C5	1.8258(13)	C5–P1–C11	108.39(6)
P1–C11	1.8317(14)	C21–P2–C27	107.45(6)
P2–C21	1.8271(14)		
P2–C27	1.8195(13)		

**Table 2:** Polymerization of 1,3-butadiene with cobalt catalytic systems.^1.^

Run	Catalyst		Polymerization				Polymer				
	Co-complex (μmol)	Al/Co	solvent	T (°C)	Time (min)	Yield (%)	1,2^2^ (%)	*rr*^3^ (%)	*M*_w_^4^ (×10^3^)	*M*_w_/*M*_n_^4^	m.p.^5^ (°C)
1	CoCl_2_(P*^t^*BuPh_2_)_2_ (10)	100	toluene	20	10	100	85.4	79.5	175.6	2.4	140.9
2	CoCl_2_(P*^t^*BuPh_2_)_2_ (30)	10	toluene	20	30	100	85.6	80.6	197.0	2.2	146.3
3	CoCl_2_(P*^t^*BuPh_2_)_2_ (30)	10	heptane	20	60	100	90.8	82.3	186.7	2.1	153.5
4	CoCl_2_(P*^t^*BuPh_2_)_2_ (30)	25	heptane	0	60	100	94.2	84.1	225.6	1.9	158.2
5	CoCl_2_[PPh_2_(NMDPP)]_2_ (10)	100	toluene	20	15	100	86.0	81.3	203.4	2.3	141.8
6	CoCl_2_[PPh_2_(NMDPP)]_2_ (20)	25	heptane	20	60	100	88.3	83.7	196.0	2.1	154.7
7	CoCl_2_[PPh_2_(NMDPP)]_2_ (30)	25	heptane	0	10	72	94.5	85.5	237.2	2.0	160.8
8	CoCl_2_(P*^i^*PrPh_2_)_2_ (5)	100	toluene	20	5	100	85.4	74.0	169.0	2.2	126.0
9	CoCl_2_(PCyPh_2_)_2_ (5)	100	toluene	20	5	75	84.5	69.0	172.0	2.4	109.0

^1^ Polymerization conditions: solvent, total volume 16 mL; butadiene, 2 mL; ^2^ percentage of 1,2 units, determined by ^1^H- and ^13^C-NMR; the remaining units are essentially *cis*-1,4, the *trans*-1,4 units being almost negligible; ^3^ percentage of syndiotactic triads, determined by ^13^C-NMR; ^4^ average molecular weight (*M*_w_, in g mol^−1^) and molecular weight distribution (*M*_w_/*M*_n_) by SEC; ^5^ melting point by DSC.

**Table 3:** Crystallographic data, data collection details, and results of refinement for complex **1**.

formula, *M*_r_	C_32_H_38_Cl_2_CoP_2_, 614.39
crystal system	Monoclinic
space group, *Z*	*P*2_1_/*n*, 4
*D*_calc_, g cm^−3^	1.347
*a*, Å	11.9782(5)
*b*, Å	15.4120(6)
*c*, Å	16.7851(7)
*β*, °	102.098(1)
*V*, Å^3^	3029.8(2)
crystal size, mm	0.35 × 0.20 × 0.08
color, habit	light blue, plate
*μ*, mm^−1^	0.868
radiation	MoK_α_
*T*, K	153(2)
2*θ*_max_, °	63.69
*h*, *k*, *l* ranges	−17→17; −22→22; −24→24
intensity decay, %	0.00
adsorption correction	multi-scan
*T*_min_, *T*_max_	0.682, 0.746
measured reflections	60180
*R*_int_	0.0320
independent reflections	9958
reflections with *I* > 2*σ*(*I*)	8422
no. of parameters	340
*R*, *wR* [*F*^2^ > 2*σ*( *F*^2^)]	0.0353, 0.0900
goodness of fit	1.048
Δ*ρ*_max_, Δ*ρ*_min_ (eÅ^−3^)	1.069, −0.231

## Supplementary Materials

Tables of atomic coordinates, anisotropic thermal parameters, bond lengths and angles of **1** may be obtained free of charge from The Director CCDC, 12 Union Road, Cambridge CB2 1 EZ, UK, on quoting the deposition numbers CCDC 1908992 the names of the authors and the journal citation (http://www.ccdc.cam.ac.uk). The following are available online: Figures S1−S6: ^13^C NMR spectra (olefinic region) of the obtained poly(1,3-butadiene)s, Figures S7−S13: FTIR spectra of the obtained poly(1,3-butadiene)s, Figures S14−S18: X-ray powder spectra of some selected poly(1,3-butadiene)s, Figure S19: FTIR spectrum of **1**, Figure S20: FTIR spectrum of **2**.
