# Supplementary material for: Novel Cobalt Dichloride Complexes with Hindered Diphenylphosphine Ligands: Synthesis, Characterization, and Behavior in the Polymerization of Butadiene
Source: Molecules. 2019 Jun 21;24(12):2308. doi: 10.3390/molecules24122308 (PMC6630202; doi:10.3390/molecules24122308)
Supplement: Supplementary file 1 [file molecules-24-02308-s001.pdf]

Supplementary Materials

Novel cobalt dichloride complexes with hindered diphenylphosphine ligands: synthesis, characterization and behavior in the polymerization of butadiene

Giovanni Ricci[1*], Giuseppe Leone[1], Ivana Pierro[1], Giorgia Zanchin[1], Alessandra Forni[2]

[1] CNR-Istituto per lo Studio delle Macromolecole (ISMAC), via A. Corti 12, 20133 Milano, Italy; giuseppe.leone@ismac.cnr.it (G.L.); ivana.pierro@ismac.cnr.it (I.P.); giorgia.zanchin@ismac.cnr.it (G.Z.)
[2] CNR-Istituto di Scienze e Tecnologie Molecolari (ISTM), via C. Golgi 19, 20133 Milano, Italy; alessandra.forni@istm.cnr.it (A.F.)
* Correspondence: giovanni.ricci@ismac.cnr.it (G.R.)

Table of Contents

Figures S1–S6. ^{13}C NMR spectra (olefinic region) of the obtained poly(1,3-butadiene)s (Table 2 in the manuscript).

Figures S7–S13. FTIR spectra of the obtained poly(1,3-butadiene)s (Table 2 in the manuscript).

Figures S14–S18. X-ray powder spectra of some selected poly(1,3-butadiene)s (Table 2 in the manuscript).

Figure S19. FTIR spectrum of $CoCl_2(P^tBuPh_2)_2$ (**1**).

Figure S20. FTIR spectrum of $CoCl_2[PPh_2(NMDPP)]_2$ (**2**).

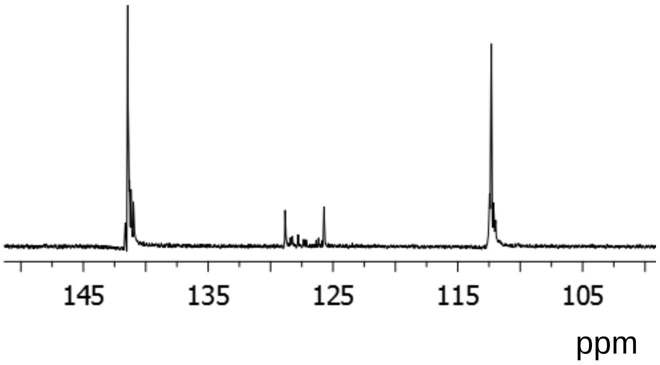

Figure S1. ^{13}C NMR spectrum (olefinic region) of the polybutadiene of Table 2, run 1.

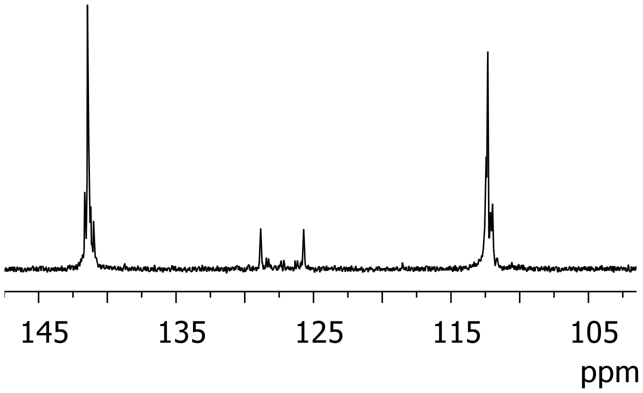

Figure S2. ^{13}C NMR spectrum (olefinic region) of the polybutadiene of Table 2, run 3.

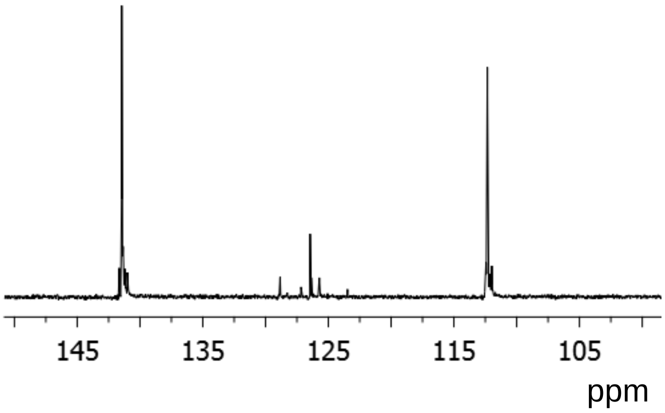

Figure S3. ^{13}C NMR spectrum (olefinic region) of the polybutadiene of Table 2, run 4.

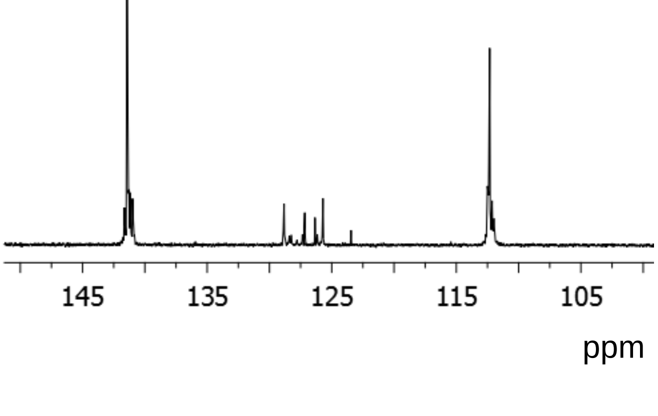

Figure 4. ^{13}C NMR spectrum (olefinic region) of the polybutadiene of Table 2, run 5.

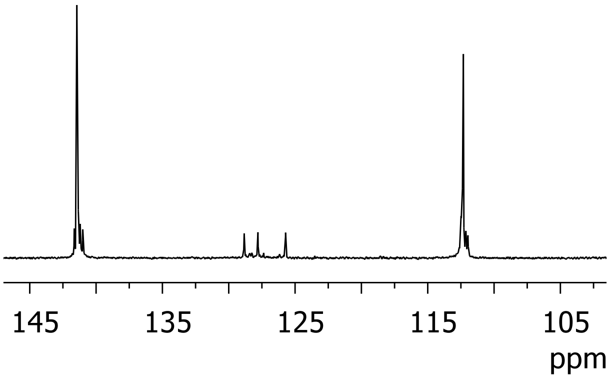

Figure 5. ^{13}C NMR spectrum (olefinic region) of the polybutadiene of Table 2, run 6.

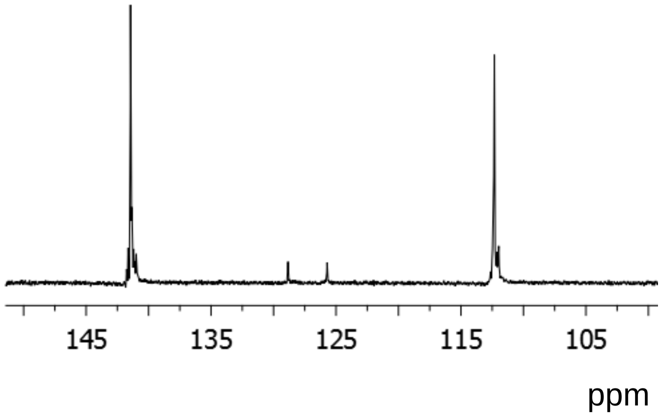

Figure 6. ^{13}C NMR spectrum (olefinic region) of the polybutadiene of Table 2, run 7.

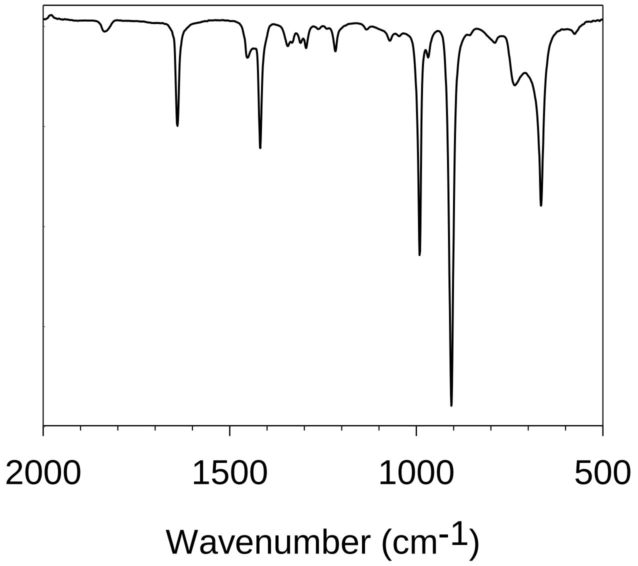

Figure 7. FTIR spectrum of the polybutadiene of Table 2, run 1.

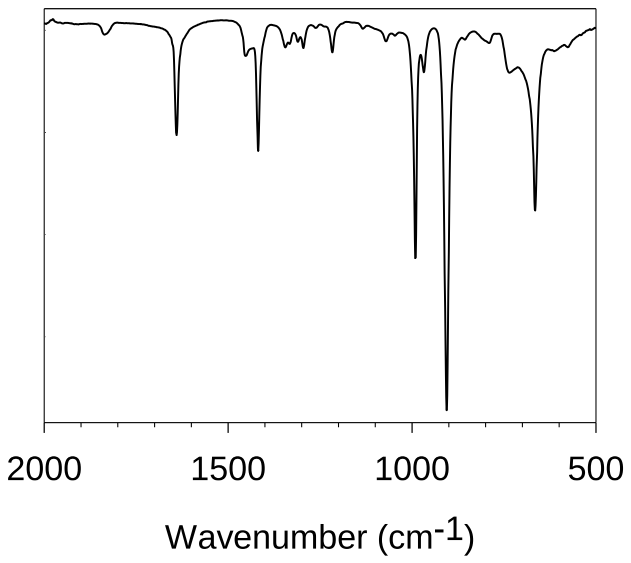

Figure 8. FTIR spectrum of the polybutadiene of Table 2, run 2.

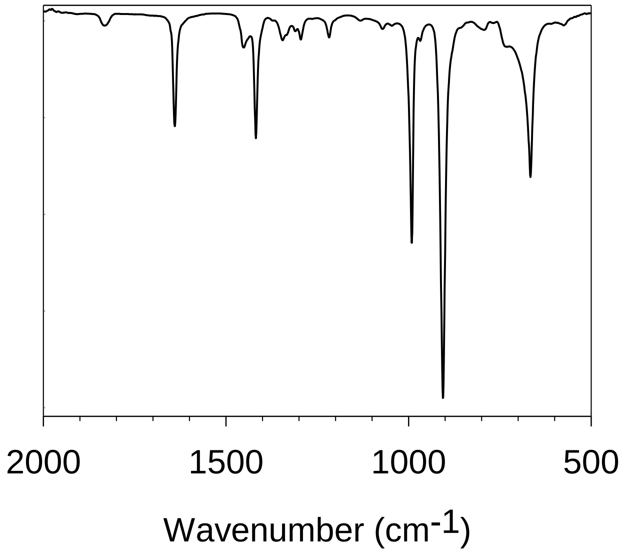

Figure 9. FTIR spectrum of the polybutadiene of Table 2, run 3.

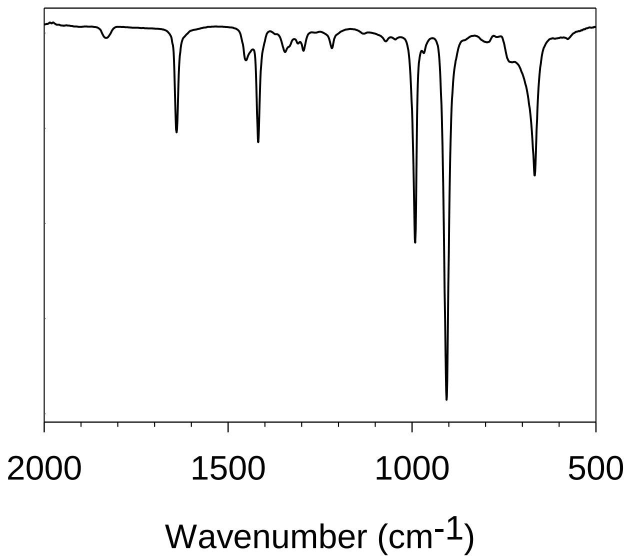

Figure 10. FTIR spectrum of the polybutadiene of Table 2, run 4.

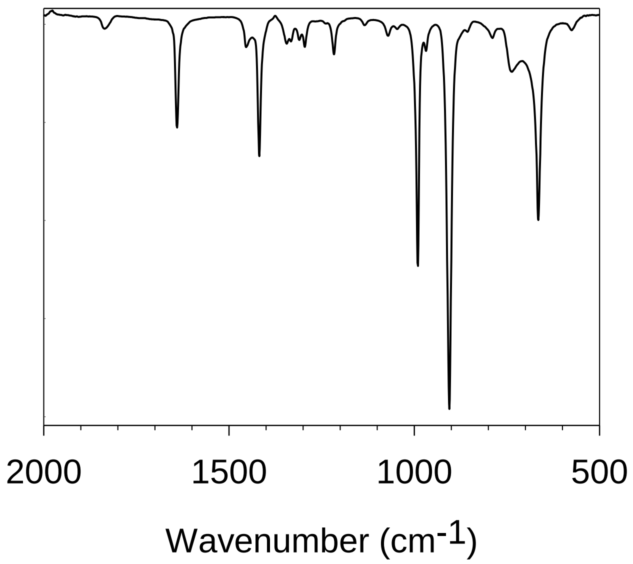

Figure 11. FTIR spectrum of the polybutadiene of Table 2, run 5.

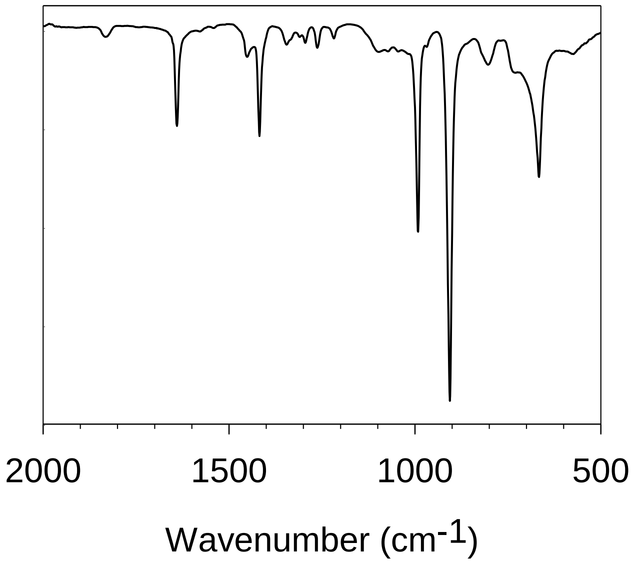

Figure 12. FTIR spectrum of the polybutadiene of Table 2, run 6.

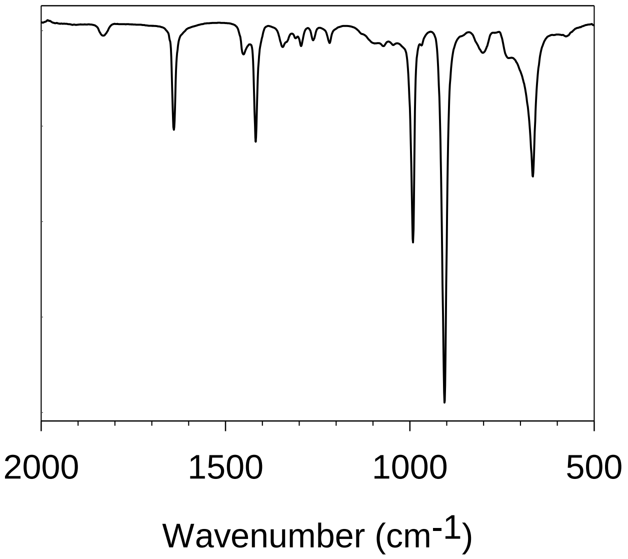

Figure 13. FTIR spectrum of the polybutadiene of Table 2, run 7.

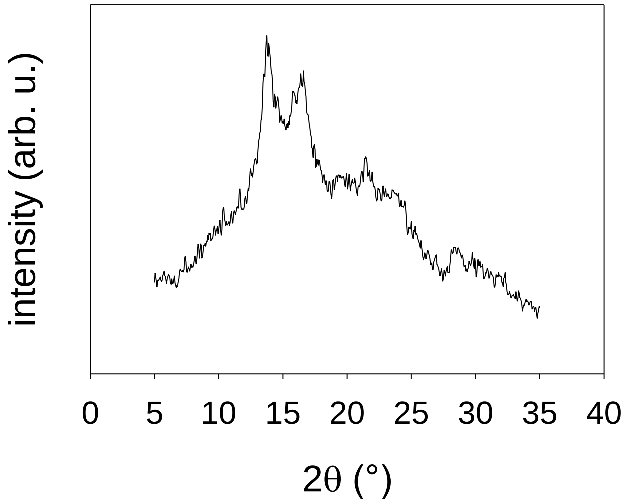

Figure S14. X-ray powder spectrum of the polybutadiene of Table 2, run 1

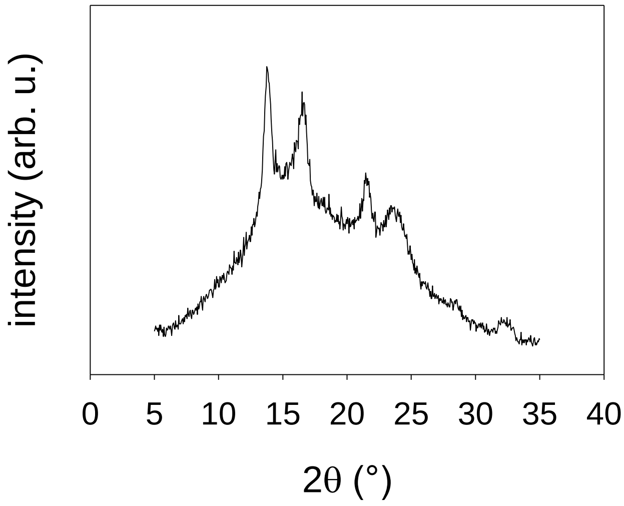

Figure S15. X-ray powder spectrum of the polybutadiene of Table 2, run 2.

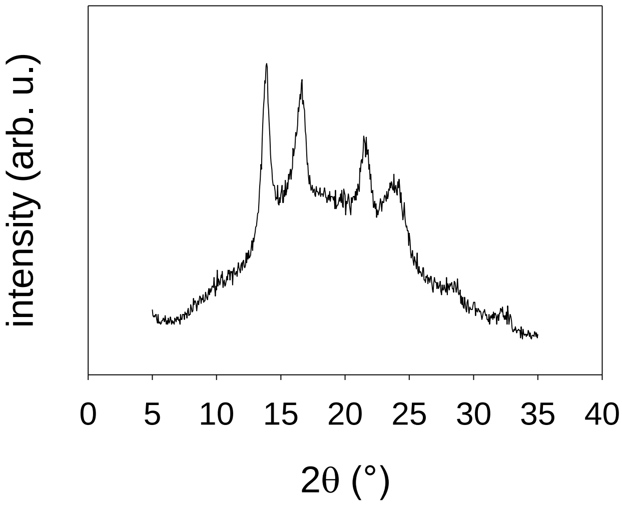

Figure S16. X-ray powder spectrum of the polybutadiene of Table 2, run 3.

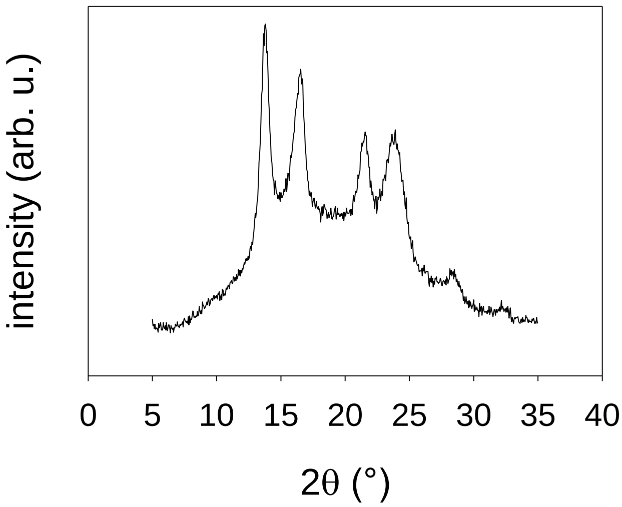

Figure S17. X-ray powder spectrum of the polybutadiene of Table 2, run 4.

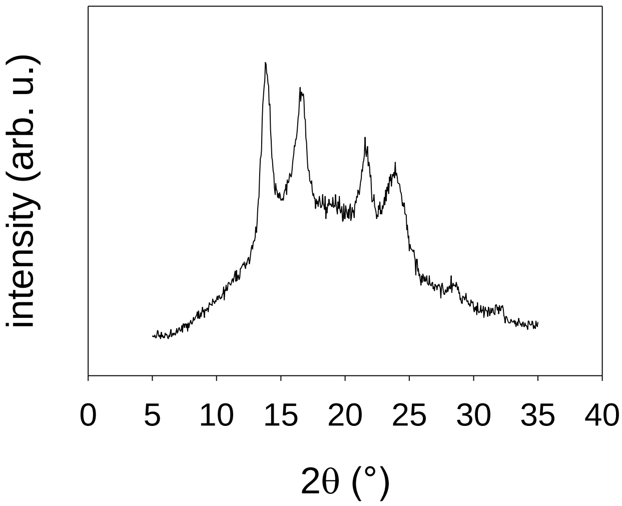

Figure S18. X-ray powder spectrum of the polybutadiene of Table 2, run 6.

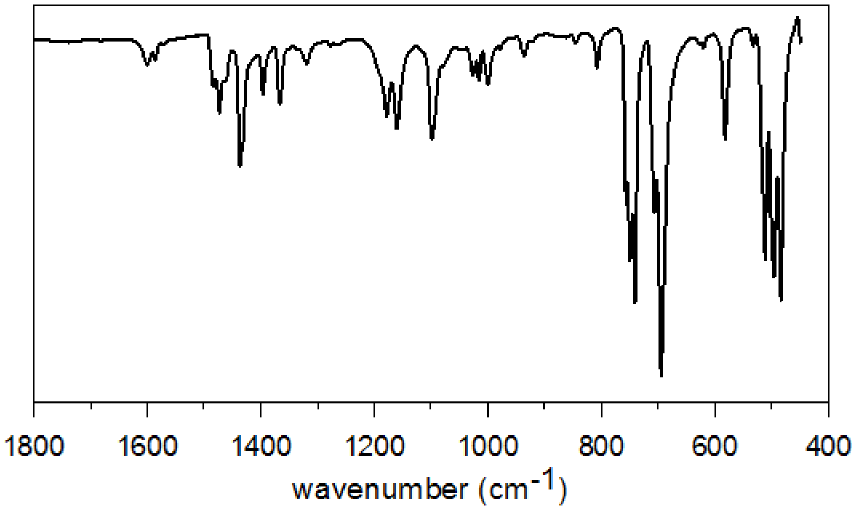

Figure S19. FTIR spectrum of $CoCl_2(P^tBuPh_2)_2$ (**1**).

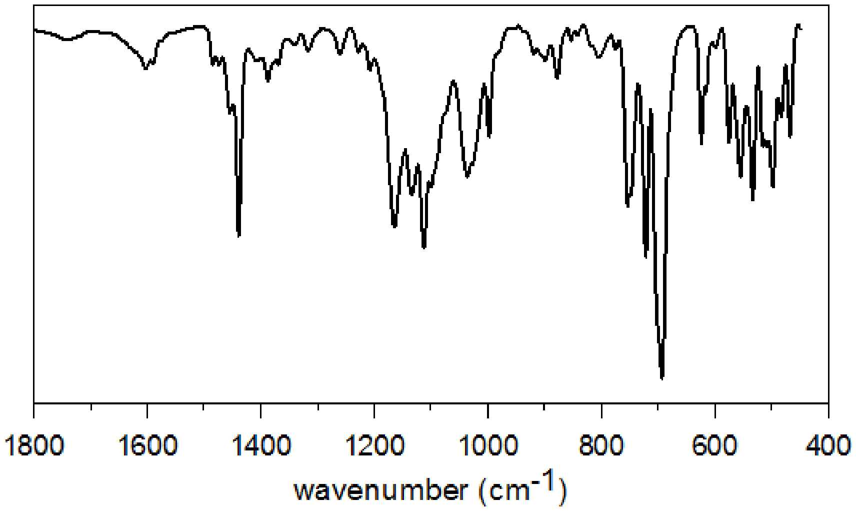

Figure 20. FTIR spectrum of $CoCl_2[PPh_2(NMDPP)]_2$ (**2**).
